# Histologic lesion type correlates of magnetic resonance imaging biomarkers in four-repeat tauopathies

**DOI:** 10.1093/braincomms/fcac108

**Published:** 2022-04-28

**Authors:** Arenn F. Carlos, Nirubol Tosakulwong, Stephen D. Weigand, Marina Buciuc, Farwa Ali, Heather M. Clark, Hugo Botha, Rene L. Utianski, Mary M. Machulda, Christopher G. Schwarz, Robert I. Reid, Matthew L. Senjem, Clifford R. Jack, J. Eric Ahlskog, Dennis W. Dickson, Keith A. Josephs, Jennifer L. Whitwell

**Affiliations:** 1 Department of Neurology, Mayo Clinic, Rochester, MN 55905, USA; 2 Department of Quantitative Health Sciences, Mayo Clinic, Rochester, MN 55905, USA; 3 Department of Psychology and Psychiatry, Mayo Clinic, Rochester, MN 55905, USA; 4 Department of Radiology, Mayo Clinic, 200 1st St SW, Rochester, MN 55905, USA; 5 Department of Information Technology, Mayo Clinic, Rochester, MN 55905, USA; 6 Department of Neuroscience, Mayo Clinic, Jacksonville, FL 32224, USA

**Keywords:** 4R tauopathy, volumetric MRI, DTI, tau lesions, biomarkers

## Abstract

Primary four-repeat tauopathies are characterized by depositions of the four-repeat isoform of the microtubule binding protein, tau. The two most common sporadic four-repeat tauopathies are progressive supranuclear palsy and corticobasal degeneration. Because tau PET tracers exhibit poor binding affinity to four-repeat pathology, determining how well *in vivo* MRI findings relate to underlying pathology is critical to evaluating their utility as surrogate markers to aid in diagnosis and as outcome measures for clinical trials. We studied the relationship of cross-sectional imaging findings, such as MRI volume loss and diffusion tensor imaging white matter tract abnormalities, to tau histopathology in four-repeat tauopathies. Forty-seven patients with *antemortem* 3 T MRI volumetric and diffusion tensor imaging scans plus post-mortem pathological diagnosis of a four-repeat tauopathy (28 progressive supranuclear palsy; 19 corticobasal degeneration) were included in the study. Tau lesion types (pretangles/neurofibrillary tangles, neuropil threads, coiled bodies, astrocytic lesions) were semiquantitatively graded in disease-specific cortical, subcortical and brainstem regions. *Antemortem* regional volumes, fractional anisotropy and mean diffusivity were modelled using linear regression with post-mortem tau lesion scores considered separately, based on cellular type (neuronal versus glial), or summed (total tau). Results showed that greater total tau burden was associated with volume loss in the subthalamic nucleus (*P* = 0.001), midbrain (*P* < 0.001), substantia nigra (*P* = 0.03) and red nucleus (*P* = 0.004), with glial lesions substantially driving the associations. Decreased fractional anisotropy and increased mean diffusivity in the superior cerebellar peduncle correlated with glial tau in the cerebellar dentate (*P* = 0.04 and *P* = 0.02, respectively) and red nucleus (*P* < 0.001 for both). Total tau and glial pathology also correlated with increased mean diffusivity in the midbrain (*P* = 0.02 and *P* < 0.001, respectively). Finally, increased subcortical white matter mean diffusivity was associated with total tau in superior frontal and precentral cortices (each, *P* = 0.02). Overall, results showed clear relationships between *antemortem* MRI changes and pathology in four-repeat tauopathies. Our findings show that brain volume could be a useful surrogate marker of tau pathology in subcortical and brainstem regions, whereas white matter integrity could be a useful marker of tau pathology in cortical regions. Our findings also suggested an important role of glial tau lesions in the pathogenesis of neurodegeneration in four-repeat tauopathies. Thus, development of tau PET tracers selectively binding to glial tau lesions could potentially uncover mechanisms of disease progression.

## Teaser

**‘**Carlos et al. studied the relationships between MRI biomarkers and tau histologic lesion types in four-repeat tauopathies and found that volume correlated with tau lesion severity in subcortical and brainstem areas, whereas tau severity in cortical areas and some key brainstem areas correlated with changes in diffusion tensor imaging metrics.’

## Graphical Abstract

**Graphical Abstract:**
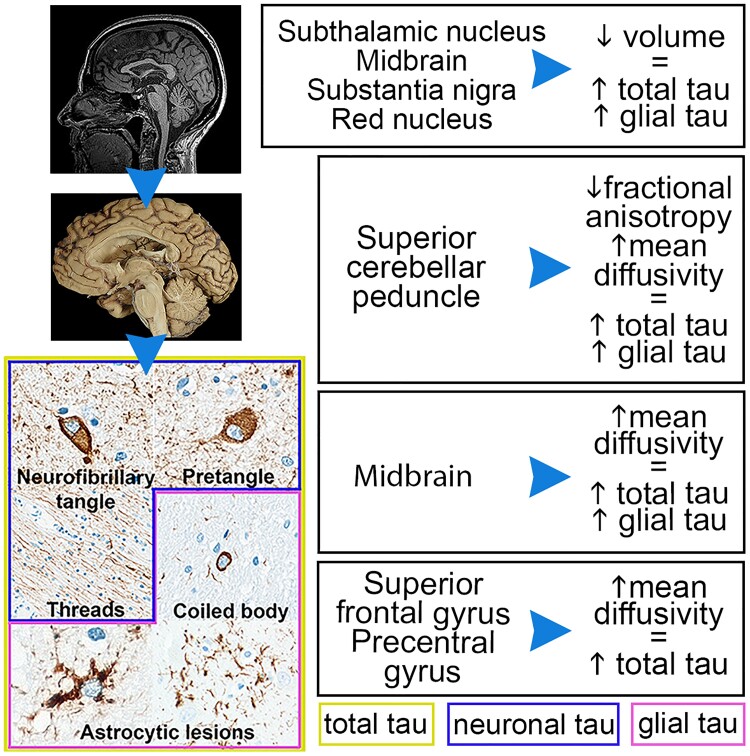
MRI biomarkers correlate with 4R tau lesion types.

## Introduction

Neurodegenerative diseases characterized by depositions of the four-repeat (4R) isoform of the microtubule-binding protein tau are referred to as primary 4R tauopathies.^1^ Two of the most common sporadic 4R tauopathies are progressive supranuclear palsy (PSP) and corticobasal degeneration (CBD), which have overlapping pathological features including tau deposits within neurons and glial cells.^2,3^ The tau neuronal and oligodendrocytic hallmarks shared by both diseases include: (i) argyrophilic neuronal cytoplasmic neurofibrillary tangles, which can be globose-shaped in PSP or densely packed in CBD when mature; when immature they are seen as diffuse and granular (also called pretangles); (ii) neuropil threads, consisting of tau deposits in neuronal axonal processes; (iii) coiled bodies, which are comma-like intracytoplasmic inclusions within oligodendrocytes. Astrocytic tau lesions are seen in two forms: (i) tufted astrocytes, containing star-like tufts of fibres in proximal processes present only in PSP; and (ii) astrocytic plaques, consisting of short, stubby tau-positive distal processes specific to CBD.^2–5^ These tau lesion types have characteristic patterns of distribution: in PSP, neurofibrillary tangles are common in the subcortical nuclei, particularly the subthalamic nucleus and substantia nigra, whereas in CBD, they are seen in cortical regions and striatum; coiled bodies are mainly found in the subcortical grey and white matter; tufted astrocytes predominate in the neocortex and the striatum; and astrocytic plaques and neuropil threads are frequent in cortical regions and in the substantia nigra.^2–6^ Of note, the brunt of the PSP pathology is in the brainstem and subcortical nuclei, whereas the cortex and basal ganglia are the primary sites of CBD pathology.^2^

Finding a radiological biomarker that correlates well with the underlying 4R tau pathology *in vivo* has been a challenge.^7^ Currently existing tau PET tracers exhibit higher binding affinity to Alzheimer-type paired helical filament-tau, consisting of three-repeat (3R) + 4R isoforms, over isolated 3R or 4R tau.^8^ Hence, the value of these tracers in 4R tauopathies is unclear. Structural MRI and diffusion tensor imaging (DTI) studies have identified a network of regional grey matter atrophy and white matter tract degeneration in 4R tauopathies.^9,10^ This structural network includes atrophy of the midbrain, striatum, globus pallidus, thalamus and frontal (premotor and motor cortex), with white matter tract degeneration of the superior cerebellar peduncle, corpus callosum, internal capsule, posterior thalamic radiations and some association tracts. Patients with PSP tend to show greater involvement of infratentorial aspects of this network, while patients with CBD show greater involvement of supratentorial aspects of this network.^9–15^ However, patterns of degeneration within this system can also vary depending on the clinical presentation.^16,17^ The relationship between these imaging findings and the actual neuropathological changes underlying these abnormalities has not yet been well characterized.

Most PSP and CBD series comparing brain MRI findings with tau burden have estimated tau deposition *in vivo* using PET imaging.^17–21^ Only a few single case reports^22–25^ or studies with about a handful of patients^26–28^ have described the relationship between MRI findings and histopathology in autopsy-confirmed PSP or CBD. Furthermore, these studies focused more on the corresponding cerebral or brainstem atrophy at autopsy and less on histology. One study has assessed the relationship with the cellular type of tau inclusions^29^ and none so far has described associations with the severity of tau lesion types in various key regions in 4R tauopathies. Moreover, studies correlating DTI metrics and pathological tau lesions are lacking. Determining how well MRI findings relate to underlying histology/lesion type is important to evaluate the utility of MRI measures as surrogate markers of pathology to aid in diagnosis and as outcome measures for clinical trials.

Our study aim was to assess the relationship of underlying tau neuropathology in disease-specific regions to the *in vivo* MRI volume loss and white matter tract abnormalities in a cohort of 47 autopsy-confirmed patients with a 4R tauopathy, including PSP and CBD. We hypothesized that the severity of imaged regional atrophy and white matter tract degeneration would correlate with tau burden, but that the strength of these relationships will vary across tau lesion types.

## Materials and methods

### Participant cohort

Our cohort consisted of research patients prospectively recruited by the Neurodegenerative Research Group at Mayo Clinic in Rochester, Minnesota between September 2009 and June 2021, who were followed up longitudinally until death and underwent autopsy with a pathological diagnosis of either PSP or CBD. All patients also underwent serial neurological examination, neuropsychological testing and a 3 T head MRI. For this study, we included patients who had completed at least one *antemortem* 3 T volumetric MRI and DTI scan using a GE scanner (GE Healthcare, Milwaukee, Wisconsin) (*n* = 47). If a patient had more than one scan, the closest one to the time of death was used.

Screening for cognitive impairment was performed using the Montreal Cognitive Assessment (MoCA) battery^30^ and motor abnormalities were assessed using the Movement Disorders Society-sponsored revision of the Unified Parkinson’s Disease Rating Scale III (MDS-UPDRS III).^31^ Disease severity and disability were evaluated using the PSP Rating Scale.^32^ This study was approved by the Mayo Clinic Institutional Board, and all patients consented to participate in the study.

### Neuropathologic evaluation and diagnosis

Following removal of the brain at autopsy, one hemisphere (typically the left) was systematically processed to yield formalin-fixed paraffin-embedded brain sections examined using Hamatoxylin and Eosin, Thioflavin-S, and Gallyas silver stain. In particular, coronal slices of the cerebral hemisphere were used to obtain sections of the superior frontal gyrus at the level of anterior hippocampus, precentral gyrus in the posterior frontal lobe, ventral thalamus and subthalamic nucleus caudal to the posterior boundary of the mammillary bodies, and striatum and globus pallidus at the level of anterior commissure. A transverse cut of the brainstem at the level of superior colliculus was used to obtain sections of the midbrain, substantia nigra and red nucleus. Finally, a transverse cut of the cerebellum perpendicular to the long axis of the brainstem was used to obtain sections of the cerebellar dentate nucleus. All cases underwent assessment of tau pathology in the abovementioned cortical, subcortical, and brainstem areas on sections immunostained with a phospho-tau monoclonal antibody (CP13; 1:1000; mouse IgG1 to phosphoserine 202, gift from the late Peter Davis, PhD, Feinstein Institute, Long Island, NY). Other concomitant pathologies such as aging-related tau astrogliopathy, argyrophilic grain disease and Alzheimer-type neurofibrillary tangles were also recorded. A Braak neurofibrillary tangle stage^33^ was assigned to each case. All neuropathological examinations were conducted by the same neuropathologist (D.W.D) with expertise in degenerative diseases to confirm that the pathological diagnoses met published criteria for PSP^34^ or CBD.^5^

### Semiquantitative 4R tau lesion burden

**Figure 1:**
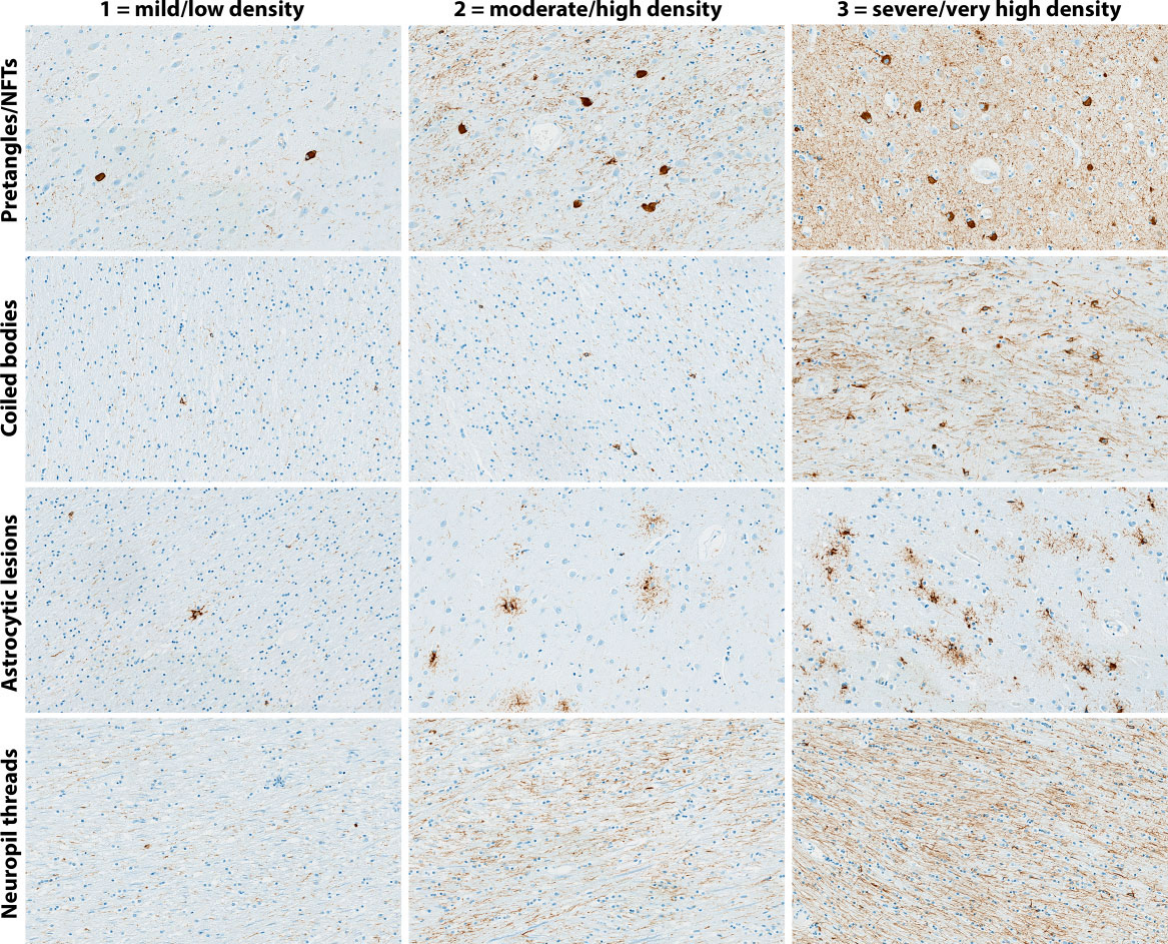
**Semiquantitative scoring of tau lesion burden.** An example of semiquantitative scoring of the different tau lesion types seen with phospho-tau immunostaining using CP13 in cases with PSP and CBD. Pretangles/NFTs, coiled bodies and astrocytic lesions (only tufted astrocytes are shown) were semiquantitatively graded as 1 = mild, 2 = moderate or 3 = severe. Neuropil threads were graded as 1 = low density; 2 = high density or 3 = very high density. Rows show the different tau lesion types. Columns show the increasing severity of pathological lesions. All images are at ×200 magnification. NFT = neurofibrillary tangles

A semiquantitative assessment of 4R tau lesion burden using slides immunostained with CP13 antibody was performed in 10 regions of interest pertinent to 4R tauopathies: superior frontal cortex, precentral (motor) cortex, striatum, globus pallidus, ventral thalamus, subthalamic nucleus, midbrain tegmentum, substantia nigra, red nucleus and cerebellar dentate nucleus.^5,34^ For each of these regions, the lesion count for pretangles/neurofibrillary tangles, coiled bodies, and tufted astrocytes (for PSP) or astrocytic plaques (for CBD)—together referred to as astrocytic lesions—was semiquantitatively graded using a four-point scale based on the number of lesions found per ×200 magnification visual field as: 0 = absent; 1 = mild (1–3 lesions); 2 = moderate (4–6 lesions); or 3 = severe (≥7 lesions). Neuropil threads were graded similarly from 0–3 for absent, low, high, or very high density. The regions of interest were assessed in their entirety and scores from individual visual fields were averaged to obtain the final score. An example of semiquantitative scoring of the different tau lesions are seen in Fig. 1.

### Volumetric MRI and diffusion tensor imaging analysis

All participants underwent serial volumetric T1-weighted MRI and DTI on a 3 T GE scanner. For the 3 T head MRI, a magnetization prepared rapid acquisition gradient echo (MPRAGE) was performed using the following parameters: TR/TE/TI, 2300/3/900 ms; flip angle 8°, 26 cm FOV; 256 × 256 in-plane matrix with a phase FOV of 0.94, and a slice thickness of 1.2 mm. The DTI acquisition consisted of a single shot echoplanar (EPI) pulse sequence with acquisition matrix 128 × 128 in the axial plane, and approximate TE 64 ms, TR = 10 s, FOV 35 cm, phase FOV = 0.66, 41 diffusion encoding directions and five non-diffusion weighted (b = 0) T2 images; slice thickness 2.7 mm (2.7 mm isotropic resolution). After denoising,^35^ the images, head motion and eddy current distortion were corrected using FSL’s eddy programme.^36–38^ We corrected for Gibbs ringing^39^ and then skull stripped the images.^40^ The Rician noise bias was then removed using the noise image from denoising.^41^ Diffusion tensors were estimated using non-linear least squares fitting and used to calculate fractional anisotropy (FA) and mean diffusivity (MD) images in dipy.^42^

To evaluate the relationship between regional tissue volume and tau pathology, we selected imaging regions of interest that matched the areas assessed at autopsy. T1-weighted MRI were tissue-class segmented using unified segmentation in SPM12^43^ with population-optimized priors and settings from the Mayo Clinic Adult Lifespan Template (MCALT).^43^ The MCALT template was normalized to each volumetric MRI using ANTs symmetric normalization,^44^ and this mapping was used to propagate several atlases. The MCALT ADIR122 atlas was used to calculate volume of the superior frontal gyrus, precentral cortex, thalamus, globus pallidus and striatum (putamen plus caudate). The Deep Brain Stimulation Intrinsic Template atlas^45^ was used to calculate volume of the substantia nigra, subthalamic nucleus and red nucleus. Finally, we used in-house developed midbrain and cerebellar dentate atlases, as previously described.^46^ For DTI, regional FA and MD were measured using the Johns Hopkins University (JHU) single-subject white matter ‘Eve’ atlas.^47^ The JHU atlas was brought into subject DTI space using ANTs as with the MCALT normalization, except that the template to subject registration used FA instead of the T1-weighted images. From the JHU atlas, we selected the following white matter tracts and regions structurally adjacent to some of the pathological regions of interest: superior frontal white matter, precentral white matter, midbrain, and superior cerebellar peduncle (analyzed in relation to both red nucleus and cerebellar dentate nuclei). Furthermore, MD was also measured in subcortical grey matter structures, including the globus pallidus, caudate/putamen, and thalamus. The FA and MD estimates were calculated as the median of the voxel values in each region to reduce the effect of partial volume contamination at region borders.

Volumes, FA, and MD were always calculated on the same hemisphere as the neuropathological studies except for when the hemispheres were sampled bilaterally at autopsy or when the laterality was unknown, in which case an average of both left and right volumes and DTI metrics was used. Using ^18^f-fluorodeoxyglucose-PET, nine CBD patients had greater involvement of the left hemisphere, five had more involvement of the right, and five had a symmetric pattern. In nine out of 14 asymmetric cases, the side with the most involvement was processed for pathological diagnosis.

### Statistical analysis

For each region, relationships between pathology and imaging findings were analyzed by fitting single-region linear regression models with log-transformed volume or DTI metrics as the response and the semiquantitative tau lesion scores as the predictors of interest using all 4R tauopathy cases. Three different models were used. In the first model, the primary predictor was total tau burden defined as the sum of the semiquantitative scores for pretangles/neurofibrillary tangles, neuropil threads, coiled bodies and astrocytic lesions. In the second model, pretangles/neurofibrillary tangles and neuropil threads were summed together to form a ‘neuronal lesions’ score, while astrocytic lesions and oligodendroglial coiled bodies were summed to form a ‘glial lesions’ score. In the third model, each of the four semiquantitative scores was included as separate predictors. All models included time from MRI scan to death and age at death as covariates. Volumetric models were also adjusted for total intracranial volume, to account for differences in head size. We used log transformations of the response for all models to facilitate comparisons across regions and modalities. We then back-transformed the regression estimates using the formula 100 × (exp(β) − 1) so that effects could be interpreted in terms of percentage differences. We used penalized maximum likelihood estimation (PMLE)^48^ to address potential issues around multiple comparisons with the goal of improving estimation accuracy rather than strictly controlling false positives at the expense of false negatives.^49^ Specifically, we used a penalty equivalent to a normal prior on the semiquantitative score coefficients specifying a 95% prior probability that a one-unit increase in a semiquantitative burden score had an effect on the mean response within ±10%. That is, β was normally distributed with mean 0 and SD 0.05. Other coefficients in the models were essentially unpenalized. The results from this PMLE approach and that of ordinary least squares (OLS) were essentially the same, except in the globus pallidus, where the PMLE estimate is likely more reliable and accurate. All statistical analyses were performed using R version 3.6 with model fitting based on the **bayesglm** function in the **arm** package.^50^ Significance level was set at alpha = 0.05.

### Data availability

Data that support the findings in this study are available from the corresponding author upon reasonable request.

## Results

### Demographic, clinical and pathological characteristics

Of the 47 patients who met our inclusion criteria, 28 (60%) had a pathological diagnosis of PSP and 19 (40%) CBD. The median age at onset was 64 years (IQR: 58–70), the median age at MRI was 70 years (65–75) and the median age at death was 73 years (67–78) (Table 1). More than two-third of our cases had presented with a typical clinical presentation specific to PSP (Richardson syndrome) or CBD (corticobasal syndrome). Co-pathologies such as age-related tau astrogliopathy (49%) and argyrophilic grain disease (40%) were common. All cases except one had a Braak neurofibrillary tangle stage of IV or lower.

**Table 1:** Characteristics of 47 participants with sporadic 4R tauopathy

Characteristics	Total (*n* = 47)
*Demographics*	
Female, *n* (%)	22 (47%)
Education, year	16 (14, 16)
Age at onset, year	64 (58, 70)
Age at death, year	73 (67, 78)
Disease duration, year	6 (4, 8)
*Neuroimaging*	
Age at MRI, year	70 (65, 75)
Age at DTI, year	70 (65, 75)
MRI to death, year	2.3 (1.2, 3.1)
DTI to death, year	2.3 (1.3, 3.1)
*Clinical data*	
Final clinical diagnosis^a^	
PSP-RS	18 (39%)
PSP-CBS	2 (4%)
PSP-F	2 (4%)
PSP-P	2 (4%)
PSP-PGF	1 (2%)
CBS	19 (41%)
BvFTD ± parkinsonism	2 (4%)
MoCA score/30	20 (15, 24)
MDS—UPDRS III score/132	60 (43, 72)
PSP rating scale score/100	54 (37, 67)
*Neuropathology*	
PSP	28 (60%)
CBD	19 (40%)
NFT positive, *n* (%)	45 (96%)
Braak NFT stage	
I	9 (20%)
II	7 (16%)
III	19 (42%)
IV	9 (20%)
V	1 (2%)
VI	0 (0%)
ARTAG positive, *n* (%)	23 (49%)
AGD positive, *n* (%)	19 (40%)

Data are shown as median (Q1, Q3) or count (%).

^a^ One patient had an ambiguous clinical presentation and was not assigned a clinical diagnosis. Autopsy later revealed PSP pathology.

AGD = argyrophilic grain disease; ARTAG = aging-related tau astrogliopathy; bvFTD = behavioural variant of frontotemporal dementia; CBD = corticobasal degeneration; CBS = corticobasal syndrome; DTI = diffusion tensor imaging; MDS-UPDRS III = Movement Disorders Society-sponsored revision of the Unified Parkinson’s Disease Rating Scale; MoCA = Montreal Cognitive Assessment; NFT = neurofibrillary tangle; PSP = progressive supranuclear palsy; PSP-F = PSP-frontal; PSP-P = PSP-parkinsonism; PSP-PGF = PSP-progressive gait freezing; PSP-RS = PSP-Richardson syndrome.

### Tau lesion type distribution and count

**Figure 2:**
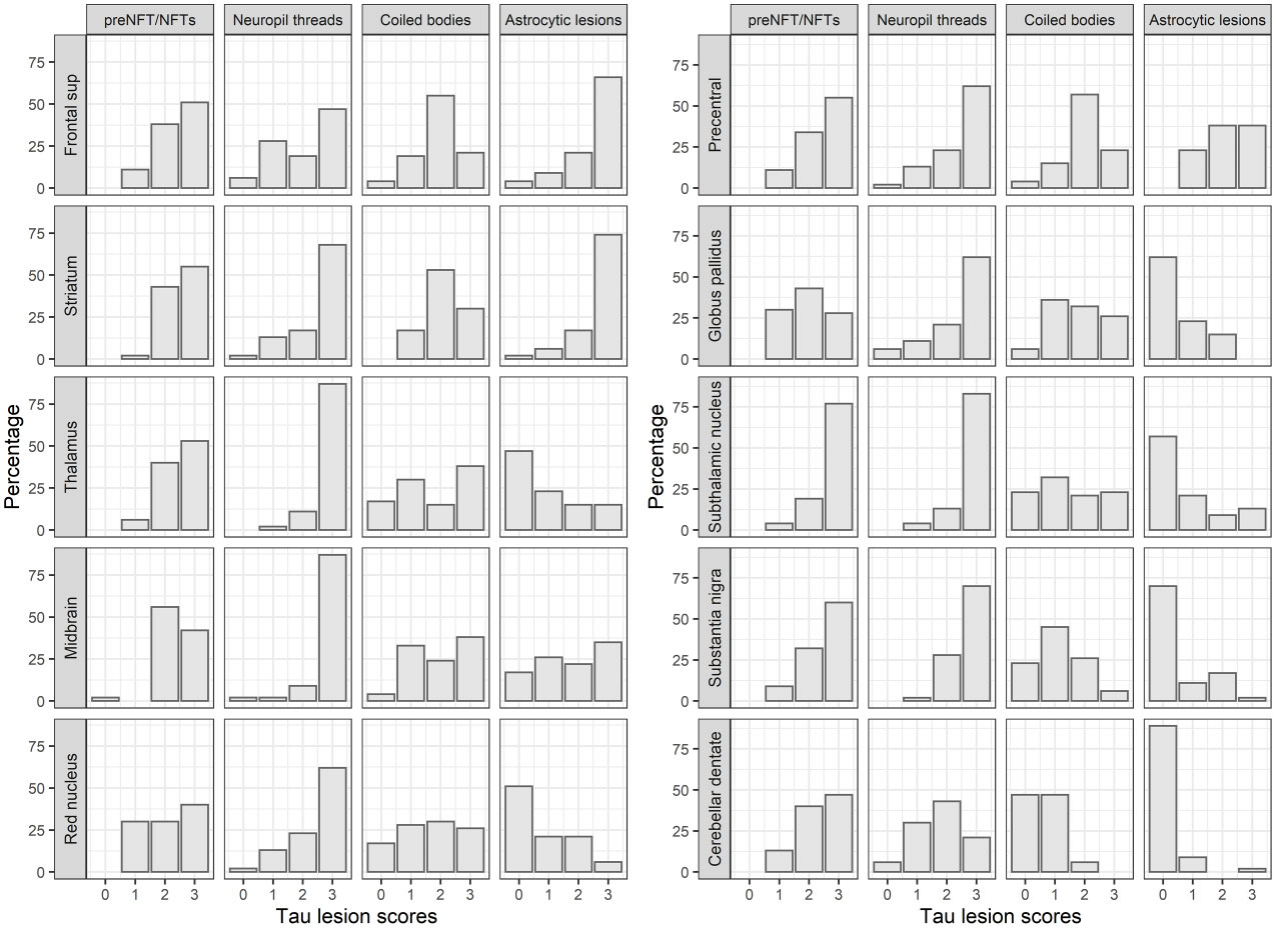
**Distribution of tau lesions counts.** Histogram plots show the distribution of the four tau lesion types across disease-specific regions in 4R tauopathies. The lesion types were semiquantitatively scored using a 4-point scale: 0 = absent, 1 = mild, 2 = moderate and 3 = severe for pretangle/neurofibrillary tangles, coiled bodies, and astrocytic lesions. For neuropil threads, the scale used was 0 = absent, 1 = low density, 2 = high density, and 3 = very high density. Frontal sup = superior frontal; NFT = neurofibrillary tangles; preNFT = pretangles

The distribution of tau lesion scores for the selected regions and all lesion types is shown in Fig. 2. Pretangles/neurofibrillary tangles commonly showed moderate or severe lesion counts in all assessed regions except for the globus pallidus and red nucleus, where mild lesion counts were as common as moderate and severe. Neuropil threads were very frequently (>75% of cases) rated as severe in the thalamus, subthalamic nucleus, and midbrain; and were often rated as severe (over 50% of cases) in precentral gyrus, striatum, pallidum, substantia nigra and red nucleus. The cerebellar dentate was the only region with mostly mild to moderate neuropil thread lesion counts. Coiled bodies were most frequently rated as moderate in the frontal and precentral cortices and striatum but showed an even distribution of lesion counts in the thalamus, subthalamic nucleus, midbrain, and red nucleus. Coiled bodies were typically absent or mild in the substantia nigra and cerebellar dentate. Astrocytic lesions were commonly found in the cortex, striatum, and midbrain, with severe lesion counts in about 75% of cases in the superior frontal cortex and striatum. Conversely, they were rarely observed in the pallidum, thalamus, subthalamic nucleus, substantia nigra red nucleus and cerebellar dentate.

### MRI regional volume loss and tau pathology

#### Volume versus total tau

**Figure 3:**
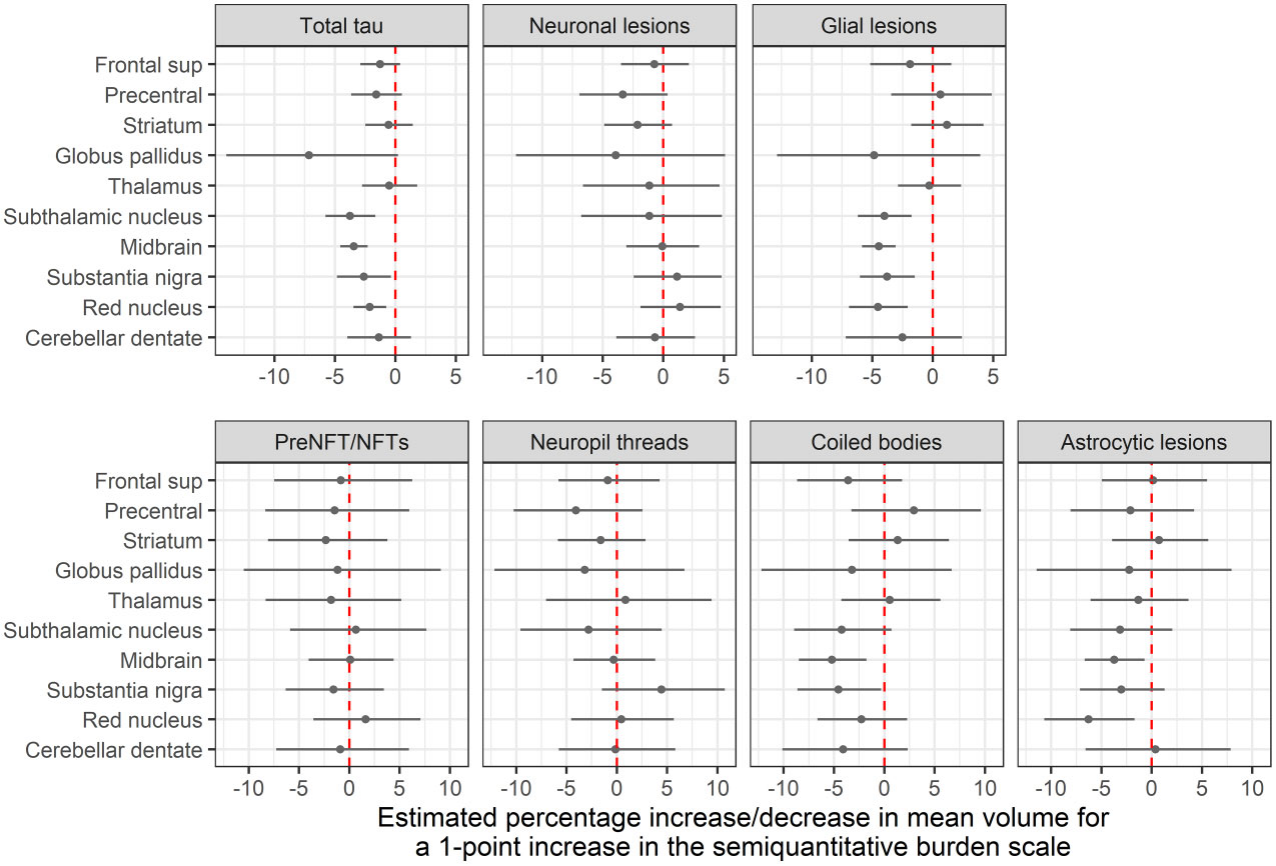
**Relationship between tau lesion scores and MRI volumes.** The forest plots display point estimates and 95% confidence intervals for the effect of a one-unit increase in semiquantitative score on volume. Estimates are expressed in terms of percentage differences and come from the PMLE approach. Confidence intervals not crossing the line of null effect (dashed vertical line) are considered significant at *P* < 0.05. A one-unit increase in total tau burden was associated with estimated 2–4% decreases in volume for the subthalamic nucleus (*P* = 0.001), substantia nigra (*P* = 0.03), red nucleus (*P* = 0.004) and midbrain (*P* < 0.001). These associations appeared to be driven by glial lesions (subthalamic nucleus, *P* = 0.001; substantia nigra, *P* = 0.003; red nucleus, *P* < 0.001; and midbrain; *P* < 0.001). Frontal sup = superior frontal gyrus; NFT = neurofibrillary tangles; preNFT = pretangle

Total tau burden (defined as sum of the semiquantitative scores across all four lesion types) was associated with antemortem volume loss in several regions. A one-unit increase in total tau burden was associated with estimated 2–4% decreases in volume for the subthalamic nucleus (*P* = 0.001), midbrain (*P* < 0.001), substantia nigra (*P* = 0.03) and red nucleus (*P* = 0.004) (Fig. 3). A one-unit increase in total tau burden in the globus pallidus trended to associate with an estimated 7% decrease in volume (*P* = 0.06). Overall, no significant associations were identified between volume and total tau burden in the superior frontal gyrus, precentral cortex, striatum, thalamus or cerebellar dentate.

#### Volume versus neuronal tau lesions

No significant associations were observed between neuronal tau lesion count and volume. In the precentral cortex, a one-unit increase in neuronal lesion count trended to associate with 3% smaller volume (*P* = 0.08).

#### Volume versus glial tau lesions

Glial lesion count was associated with volume loss in the subthalamic nucleus (*P* = 0.001), midbrain (*P* < 0.001), substantia nigra (*P* = 0.003) and red nucleus (*P* < 0.001), where a one-unit increase in the glial lesion score was associated with 4–5% decrease in volume. When astrocytic and oligodendrocytic lesions were analyzed separately, the effects were generally concordant. Still, there were differences in significance. Higher scores of coiled bodies in the substantia nigra (*P* = 0.04), and both coiled bodies and astrocytic lesions in the midbrain (*P* = 0.005 and *P* = 0.02, respectively) were associated with volume loss. Smaller volume was seen in relation to higher astrocytic burden in the red nucleus (*P* = 0.01).

### Diffusion tensor imaging-fractional anisotropy and tau pathology

#### Fractional anisotropy versus total tau

**Figure 4:**
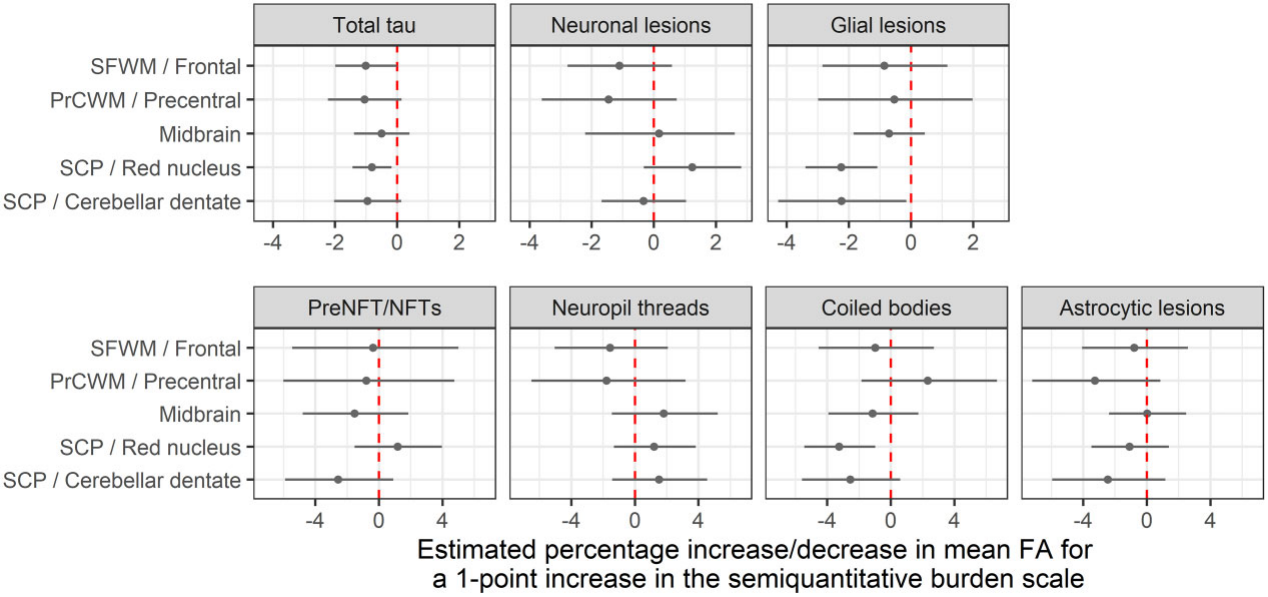
**Relationship between tau lesion scores and DTI-FA.** The forest plots display point estimates and 95% confidence intervals for the effect of a one-unit increase in semiquantitative score on fractional anisotropy (FA). Estimates are expressed in terms of percentage differences and come from the PMLE approach. Confidence intervals not crossing the line of null effect (dashed vertical line) are considered significant at *P* < 0.05. A one-unit increase in total tau burden of the red nucleus was associated with ∼1% lower FA in the superior cerebellar peduncle (*P* = 0.02). Furthermore, a one-unit greater glial lesion count in the red nucleus (*P* < 0.001) and cerebellar dentate (*P* = 0.04) were associated with ∼2% reduction in FA in the superior cerebellar peduncle, with the association in the red nucleus apparently driven by coiled bodies (*P* = 0.008). DTI = diffusion tensor imaging; FA = fractional anisotropy; NFT = neurofibrillary tangle; PrCWM = Precentral white matter; preNFT = pretangles; SCP = superior cerebellar peduncle; SFWM = superior frontal white matter.

Associations between tau lesions and FA are shown in Fig. 4. A one-unit increase in total tau burden of the red nucleus was associated with ∼1% lower FA in the superior cerebellar peduncle (*P* = 0.02). There was also a trend for an association with greater tau burden in the cerebellar dentate (*P* = 0.09). In cortical areas, only a trend for higher total tau in the overlying cortex was seen in relation with lower FA in the frontal white matter (*P* = 0.054) and precentral white matter (*P* = 0.09). No significant associations were observed between tau lesion counts and FA in the midbrain.

#### Fractional anisotropy versus neuronal lesions

No clear associations were found between neuronal lesions (whether considered in combination or individually) and FA.

#### Fractional anisotropy versus glial lesions

When glial lesions were analyzed separately, a one-unit greater lesion count in the red nucleus (*P* < 0.001) and cerebellar dentate (*P* = 0.04) were associated with ∼2% reduction in FA in the superior cerebellar peduncle. In particular, the association in the red nucleus was apparently driven by coiled bodies (*P* = 0.008).

### Diffusion tensor imaging-mean diffusivity and tau pathology

#### Mean diffusivity versus total tau

**Figure 5:**
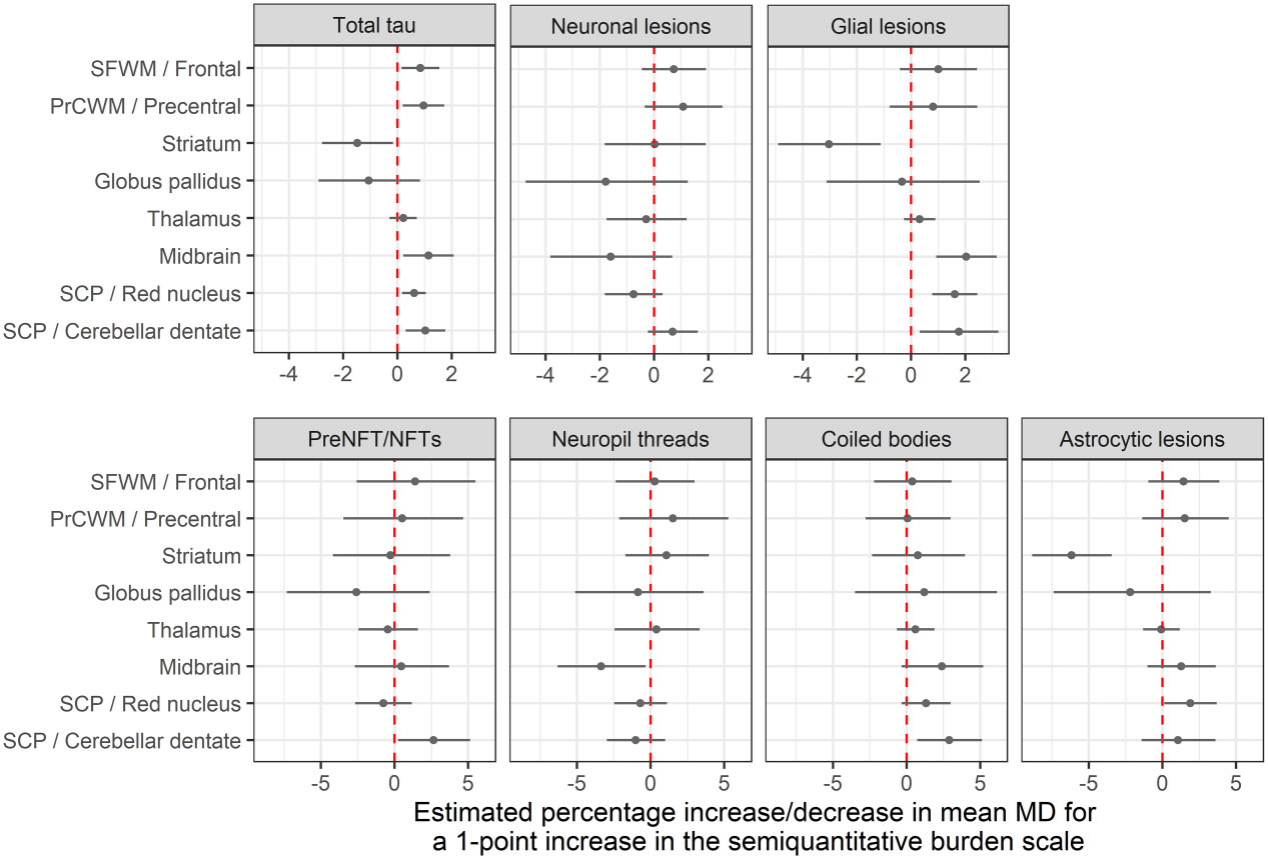
**Relationship between tau lesion scores and DTI-MD.** The forest plots display point estimates and 95% confidence intervals for the effect of a one-unit increase in semiquantitative score on mean diffusivity (MD). Estimates are expressed in terms of percentage differences and come from the PMLE approach. Confidence intervals not crossing the line of null effect (dashed vertical line) are considered significant at *P* < 0.05. About 1% higher MD in the SCP was seen with greater total tau lesion scores in both red nucleus and cerebellar dentate (each, *P* = 0.008). In the midbrain, a higher total tau burden also associated with ∼1% increased MD (*P* = 0.02). In the striatum, the effect was in the opposite direction (*P* = 0.03). Glial pathology was the main driver of the abovementioned associations. Total tau lesion scores in the superior frontal gyrus and premotor cortex were associated with ∼1% increased MD in the underlying white matter (*P* = 0.020 for both superior frontal and precentral). DTI = diffusion tensor imaging; MD = mean diffusivity; NFT = neurofibrillary tangle: PrCWM = precentral white matter; preNFT = pretangles; SCP = superior cerebellar peduncle; SFWM = superior frontal white matter.

The relationships between tau lesions and MD are shown in Fig. 5. About 1% higher MD in the superior cerebellar peduncle was associated with greater total tau lesion score in both the red nucleus (*P* = 0.008) and cerebellar dentate (*P* = 0.008). Similarly, in the midbrain, higher total tau severity also associated with ∼1% increased MD (*P* = 0.02). In the striatum, the effect was in the opposite direction (*P* = 0.03). Regarding cortical areas, total tau lesion scores in the superior frontal gyrus and premotor cortex were associated with ∼1% increased MD in the underlying white matter (*P* = 0.02 for superior frontal and *P* = 0.02 for precentral). No significant associations were observed between total tau and MD in the thalamus or globus pallidus.

#### Mean diffusivity versus neuronal lesions

Significant associations between pretangles/neurofibrillary tangles in the cerebellar dentate and higher MD (3% increase) in the superior cerebellar peduncle was found (*P* = 0.04). On the contrary, very high density neuropil threads in the midbrain were associated with a 3% decrease in MD (*P* = 0.04).

#### Mean diffusivity versus glial lesions

Associations with glial lesions generally mirrored those with total tau burden. Increased glial lesion count in the red nucleus and cerebellar dentate clearly associated with ∼2% higher MD in the superior cerebellar peduncle (*P* < 0.001 for red nucleus and *P* = 0.02 for cerebellar dentate). Glial pathology also appeared to drive the associations in the midbrain (*P* < 0.001) and striatum (*P* = 0.003). When individual glial lesions were assessed, coiled bodies in the cerebellar dentate and astrocytic lesions in the red nucleus apparently drove the significant associations (*P* = 0.01 and *P* = 0.04, respectively). In the midbrain, coiled bodies trended to associate with higher MD (*P* = 0.09), while in the striatum, high astrocytic count was associated with lower MD (*P* < 0.001).

## Discussion

In the present study, we assessed the relationship between 4R tau lesions at autopsy and *antemortem* MRI volume loss and white matter tract degeneration in sporadic 4R tauopathies. We showed that volume correlated with tau burden in subcortical and brainstem areas, in particular the basal ganglia and the mesencephalon, respectively. We also identified correlations between tau burden and changes on DTI, including a relationship between integrity of the superior cerebellar peduncle and tau burden in the red nucleus and cerebellar dentate, as well as local correlations within cortical regions, brainstem, and striatum. Thus, structural MRI and DTI measures of these regions within the 4R tauopathy network show potential as biomarkers of underlying pathology.^7^ A key factor in these relationships was glial pathology, suggesting that glial lesions are particularly important determinants of structural damage in 4R tauopathies.

Because 4R tauopathies, similar to any other neurodegenerative disease, require a pathological examination to achieve a definite diagnosis, biomarkers reflecting post-mortem pathology *in vivo* are needed. An ideal neuroimaging biomarker would typically correlate well with both the presence and the severity of the pathological burden.^7^ One of our key findings was the association between post-mortem total tau lesion score and *antemortem* MRI volume in the subthalamic nucleus, substantia nigra, red nucleus and midbrain. Previous studies have found associations between MRI volume and tau PET uptake using flortaucipir (^18^F-AV1451) in the cerebellar dentate, red nucleus and subthalamic nucleus,^19^ basal ganglia^20^ and in the cortex^18,20^ in clinically diagnosed 4R tauopathies. Although providing supportive evidence suggesting a relationship between volume and tau burden, these studies only used tau PET tracers of which the pathological specificity is uncertain. Our findings provide a direct link between volume of certain structures and underlying tau pathology and confirm the role of structural MRI as a biomarker of tau pathology in 4R tauopathies. We did not detect any correlations between volume and tau lesion score in cortical regions. It is possible that other measures such as cortical thickness, which has been shown to be efficient in detecting cortical involvement in corticobasal syndrome (CBS) and clinically diagnosed PSP,^51,52^ could have yielded more significant results. However, a recent study has shown that neither cortical volume nor thickness clearly outperformed the other, but instead showed similar results.^53^ On the contrary, we did find that greater total tau lesion scores in the cortical regions correlated with greater white matter MD and lower white matter FA. Hence, DTI measures from the white matter in the superior frontal and precentral cortex may be more sensitive than volume to underlying tau burden. This concurs with the fact that 4R tauopathies strongly target the white matter.^54^ We also assessed if the strength of the imaging-pathological correlations varied by the cellular type of tau lesion, neuronal versus glial. We found that the severity of glial tau lesions, more so than neuronal lesions, correlated with MRI volume in the subthalamic nucleus, substantia nigra, red nucleus and midbrain. In these areas, with the exclusion of the red nucleus, oligodendroglial lesions (*i.e.*, coiled bodies) were the lesion type primarily driving the associations, as astrocytic lesions were mainly absent or mild. Conversely, astrocytic pathology was more influential in the midbrain and red nucleus. We did not find any significant associations between neuronal tau and volume. One previous study assessed the relationships of neuronal and glial pathology to volume in 4R tauopathies,^29^ and showed that neuronal tau burden predicted volume across regions in PSP, whereas glial pathology trended to associate with volume in CBD. This previous study did not, however, assess relationships specifically within different brain regions and the authors included other types of tau lesions, such as neuronal cytoplasmic inclusions and thorny astrocytes, and further divided threads into cortical neuropil and white matter threads. We did not examine relationships separately in PSP and CBD due to the complexity of our analyses and the large number of regional associations already assessed in the study. Future studies investigating the disease-specific or regional-specific vulnerability of neuronal and glial cells would shed light on possible diverging pathomechanisms.

The distinction between neuronal and glial pathology is gaining more interest in the field of 4R tauopathies because of their different anatomical distribution, histopathology and pattern of spread.^55–57^ Recently, Kovacs *et al*.^55^ found that neuronal loss in PSP correlated with total tau load only in regions where neuronal tau predominated (*i.e.*, subcortical regions and brainstem) and not in cortical areas were astroglial tau were more abundant. In CBD, Ling *et al.*^58^ found that astroglial lesions were the first and most abundant type of lesion in the cortex in early stages which were later substituted by neuronal lesions as the disease progressed and neuronal loss increased. Our findings are similar as we did not find any significant correlation between total tau lesion count and volume in the frontal cortex and striatum where astrocytic lesion densities were highest. In the precentral cortex, neuronal tau trended to associate with volume concomitantly in the presence of lesser astrocytic burden. This could suggest a potentially protective role of astrocytic lesions against neurodegeneration caused by neuronal inclusions. A study by Allen *et al*.^56^ revealed that neuronal and astrocytic lesions were in fact related to contrasting transcriptional changes. In PSP, the authors found that while neuronal lesions positively correlated with expression of synaptic gene levels, either because of neurodegeneration or as a cause of increased vulnerability, astrocytic lesions were related to upregulation of the microglial transcripts and downregulation of synaptic genes. It was further suggested that upregulation of microglial transcriptional network might provide protection against formation of neurofibrillary tangles. Whether astroglial pathology provides protection against neurodegeneration remains unanswered. While previous studies had suggested that the appearance of glial inclusions was a late phenomenon^59^ occurring after axonal damage has already caused tau ‘seeding’,^60^ a recent study by Narasimhan *et al.*^57^ has suggested that oligodendrocytes are able to intrinsically produce tau, harbour pathological inclusions, and propagate it through their cellular processes independently of neuronal tau. All these were found to eventually lead to oligodendrocyte loss. Since oligodendrocytes are known to provide structural, functional and trophic support to neurons,^61^ it would be reasonable to say that oligodendrocytic cell loss combined with their ability to propagate tau can both potentially contribute to neurodegeneration and atrophy. On the other hand, astrocytic pathology is believed to be caused by neuronal-to-astrocytic tau propagation and is thus dependent on neuronal pathology.^62^ In addition, astrocytes appear to be unable to propagate tau.^57^ The possibility that astrocytes only accumulate tau inclusions secondary to neuronal unloading of tau burden would partially explain why we detected no association between neuronal tau and volumes in regions where astrocytic pathology were highest (cortex and striatum). In this theory, astrocytic pathology would likely confer protection against neurodegeneration through yet-to-be-discovered mechanisms.

As for the DTI changes, degeneration of the dentatorubrothalamic tract is now being consistently recognized as a disease-specific characteristic of PSP which could potentially be useful for clinical diagnosis and research purposes.^13,19,63,64^ The dentatorubrothalamic tract originates from the cerebellar dentate nucleus, decussates to the contralateral red nucleus through the superior cerebellar peduncle and terminates in the contralateral ventrolateral nucleus of the thalamus. According to Ishizawa *et al*.,^65^ demyelination, microgliosis and tau pathology, particularly of the oligodendroglial type, contribute to the degeneration of the dentatorubrothalamic tract. Of note, atrophy of the superior cerebellar peduncle was already part of the original description of PSP by Steel *et al.*^66^ Studies since then have revealed it as a pathognomonic sign of PSP.^14,19,67^ More recently, imaging techniques have shown that both white matter abnormalities, as signalled by decreased FA and increased MD, and reduced structural MRI volumes correlated with increased flortaucipir uptake in the cerebellar dentate and red nucleus.^19^ Our findings parallel the results of this study, but with the advantage of studying post-mortem tau deposition, as we found associations between the same abnormalities in DTI metrics and dense tau glial inclusions within the cerebellar dentate and red nucleus. This further strengthens evidence for superior cerebellar peduncle degeneration as a disease-specific biomarker. However, it remains curious why astrocytic pathology correlated with MRI volume or DTI changes only in the mesencephalon.

Grey and white matter degeneration of the thalamus is also a common finding in PSP^15,17,67,68^ and CBD.^69,70^ Thalamic degeneration was often described in relation to neurofibrillary tangle burden in PSP.^66,71,72^ Although we found moderate to severe tau burden particularly of the neuronal type in the thalamus, neither the severity of tau inclusions nor the lesion type appeared to be involved in the regional changes in volume or diffusivity. This is in line with a previous report which failed to find any correlation between flortaucipir uptake and volume loss in the thalamus.^19^ Using multimodal neuroimaging studies, thalamocortical dysfunction correlated instead with the degeneration of the dentatorubrothalamic tract, especially the superior cerebellar peduncle, and cortical atrophy.^13^ Efferents from the thalamus project to the premotor cortex and impairment of functional connectivity between thalamus and frontal cortex has already been reported.^13,19,73^ Whether this disruption is a secondary phenomenon related to intrathalamic dysfunction in turn caused by the degeneration of the dentatorubrothalamic tract or due to a primary degeneration of the frontal cortex is still unknown.^13^ While we cannot address this with certainty, we did find that severe total tau lesion scores in the superior frontal and precentral cortices correlated with increased MD and lower FA in the underlying white matter. Taken together, our findings suggest that the thalamic degeneration is probably related more to the degeneration of the dentatorubrothalamic tract, as other studies using multimodal neuroimaging techniques have demonstrated,^13,14,19^ related to either downstream or upstream pathology or both rather than tau burden in the thalamus itself.

Intriguingly, we found a strong association between high astrocytic burden and decreased MD within the striatum. Mean diffusivity quantifies cellular and membrane density^74^ and one would typically expect to see an *increase* in MD in relation to neurodegeneration. The striatum is the region affected the earliest and predominantly by astrocytic pathology in both PSP and CBD, with neuronal lesions eventually increasing in frequency as the disease progresses.^55,58^ We did find the striatum to be the region with highest percentage (about 75%) of cases with severe astrocytic burden. Hence, the possible protective role played by astrocytic tau lesions against neurodegeneration could explain this finding. On the other hand, another possible explanation for this finding is increased cellularity caused by gliosis. Microglial activation was found to parallel neurodegeneration in PSP and CBD, mostly in association with glial tau pathology.^56,65,75^ As previously mentioned, astrocytic pathology is capable of activating microglial genes in PSP.^56^ Still in PSP, Malpetti *et al*.^76^ showed that baseline neuroinflammation, as measured by PET imaging using [^11^C]PK11195 ligand to trace microglial activation, showed correlation with regional atrophy and disease progression. While no study has yet reported decreased MD in association with gliosis in PSP or CBD, similar changes were found in preclinical cases of Huntington’s disease^77^ and Alzheimer’s disease^78^ in which the decrease in MD correlated with early microglial activation. However, in our study, we selected MRI scans closest to death and hence our cohort was in the end stage of the disease. Future studies can be directed to track the progression of gliosis in both early- and end-stage PSP or CBD and its relationship with DTI metrics.

Overall, the differences in anatomical distribution and burden of glial and neuronal lesions should be considered in the production of surrogate tau tracers in 4R tauopathies. An ideal tau tracer for screening for 4R tauopathies should be able to bind to *both* glial and neuronal lesions. In a very recently published paper, our group found that flortaucipir uptake did not correlate with a specific type of tau lesion in 4R tauopathies, demonstrating no differential affinity.^79^ Alternatively, tracers that preferentially bind to either neuronal or glial tau lesions could potentially differentiate PSP from CBD based on the topography and magnitude of uptake.

The strength of this study is the selective inclusion of only pathologically confirmed cases of PSP and CBD and the large number of patients included in our study with standardized neuroimaging. Another strength is the fact that all pathological analyses were performed by the same expert neuropathologist (D.W.D.). However, our cohort included more PSP patients than CBD patients which could have had limited range and power to detect associations in some CBD disease-specific regions, especially cortical ones. Other measures of cortical involvement like cortical thickness can also be used in future studies. In addition, because CBD is mostly asymmetric and the left hemisphere was routinely sampled with the exception of a few cases, another limitation of the study was the failure to constantly sample at autopsy the hemisphere with more pathological involvement as signalled by ipsilateral hypometabolism on ^18^f-fluorodeoxyglucose-PET or by the more significant involvement of the contralateral limbs. Furthermore, our study assessed regional correlations across all 4R tauopathy cases to increase statistical power and to increase tau lesion score range within each region (*i.e.*, within PSP, all cases may have frequent inclusions in the subthalamic nucleus, for example). However, this study design limits our ability to assess disease-specific relationships. Our cohort was also clinically heterogeneous which could have influenced the findings since different clinical variants are associated with different distributions of tau pathology. Finally, an absolute match between the imaging and pathological regions of interest was unfeasible. It is important to keep in the mind that post-mortem neuropathologic studies are not only limited in time, as they only provide a cross-sectional glimpse of the changes present at the time of death, but they are also limited by the tissue samples, since it is impracticable to study a region of interest in its entirety and thin sections representative of the whole regions are instead used. This could have led to imperfect matching between the imaging and pathological regions of interests, particularly when concerning big regions, such as the cortical areas, where we did not find significant correlations between volume and tau pathology. Nevertheless, the relatively unchanged estimates obtained after penalization suggest that our findings are quite robust.

In summary, the effectiveness of volumetric MRI versus DTI changes to reflect underlying tau pathology varies by region. In subcortical and brainstem regions, tau pathology is accompanied by volume loss easily detectable by volumetric MRI. In addition, degeneration of the midbrain white matter and the superior cerebellar peduncle associated with glial tau pathology can be detected by DTI. On the other hand, in cortical regions, it appears that tau pathology is better reflected by changes in the adjacent white matter making DTI more suitable than volumetric MRI in detecting pathology. Most of the significant associations we found in this study by terms of oligodendrocytic or astrocytic lesions highlight the crucial role played by glial pathology in the pathogenesis of 4R tauopathies. Generating tau PET tracers able to specifically, or even preferentially, bind to glial tau lesions could potentially uncover the mechanisms of disease progression. Overall, our results show clear dose-response relationships between MRI/DTI changes and pathology in 4R tauopathies.

## Abbreviations

4R tauopathies =: four-repeat tauopathies
CBD =: corticobasal degeneration
DTI =: diffusion tensor imaging
FA =: fractional anisotropy
MCALT =: Mayo Clinic Adult Lifespan Template
MD =: mean diffusivity
MDS-UPDRS III =: Movement Disorders Society-Sponsored revision of the Unified Parkinson’s Disease Rating Scale
MoCA =: Montreal Cognitive Assessment
PMLE =: Penalized Maximum Likelihood Estimation
PSP =: progressive supranuclear palsy
